# Deep learning approach using SPECT-to-PET translation for attenuation correction in CT-less myocardial perfusion SPECT imaging

**DOI:** 10.1007/s12149-023-01889-y

**Published:** 2023-12-28

**Authors:** Masateru Kawakubo, Michinobu Nagao, Yoko Kaimoto, Risako Nakao, Atsushi Yamamoto, Hiroshi Kawasaki, Takafumi Iwaguchi, Yuka Matsuo, Koichiro Kaneko, Akiko Sakai, Shuji Sakai

**Affiliations:** 1 Department of Health Sciences, Faculty of Medical Sciences, Kyushu University, Fukuoka, Japan; 2 Department of Diagnostic Imaging and Nuclear Medicine, Tokyo Women’s Medical University, 8–1 Kawada-cho, Shinjuku-Ku, Tokyo, 162–8666 Japan; 3 Department of Radiology, Tokyo Women’s Medical University, Tokyo, Japan; 4 Department of Cardiology, Tokyo Women’s Medical University, Tokyo, Japan; 5 Department of Advanced Information Technology, Faculty of Information Science and Electrical Engineering, Kyushu University, Fukuoka, Japan

**Keywords:** ^13^N ammonia PET, Cardiac SPECT, Myocardial perfusion imaging, Attenuation correction, Deep learning

## Abstract

**Objective:**

Deep learning approaches have attracted attention for improving the scoring accuracy in computed tomography-less single photon emission computed tomography (SPECT). In this study, we proposed a novel deep learning approach referring to positron emission tomography (PET). The aims of this study were to analyze the agreement of representative voxel values and perfusion scores of SPECT-to-PET translation model-generated SPECT (SPECT_SPT_) against PET in 17 segments according to the American Heart Association (AHA).

**Methods:**

This retrospective study evaluated the patient-to-patient stress, resting SPECT, and PET datasets of 71 patients. The SPECT_SPT_ generation model was trained (stress: 979 image pairs, rest: 987 image pairs) and validated (stress: 421 image pairs, rest: 425 image pairs) using 31 cases of SPECT and PET image pairs using an image-to-image translation network. Forty of 71 cases of left ventricular base-to-apex short-axis images were translated to SPECT_SPT_ in the stress and resting state (stress: 1830 images, rest: 1856 images). Representative voxel values of SPECT and SPECT_SPT_ in the 17 AHA segments against PET were compared. The stress, resting, and difference scores of 40 cases of SPECT and SPECT_SPT_ were also compared in each of the 17 segments.

**Results:**

For AHA 17-segment-wise analysis, stressed SPECT but not SPECT_SPT_ voxel values showed significant error from PET at basal anterior regions (segments #1, #6), and at mid inferoseptal regions (segments #8, #9, and #10). SPECT, but not SPECT_SPT_, voxel values at resting state showed significant error at basal anterior regions (segments #1, #2, and #6), and at mid inferior regions (segments #8, #9, and #11). Significant SPECT overscoring was observed against PET in basal-to-apical inferior regions (segments #4, #10, and #15) during stress. No significant overscoring was observed in SPECTSPT at stress, and only moderate over and underscoring in the basal inferior region (segment #4) was found in the resting and difference states.

**Conclusions:**

Our PET-supervised deep learning model is a new approach to correct well-known inferior wall attenuation in SPECT myocardial perfusion imaging. As standalone SPECT systems are used worldwide, the SPECT_SPT_ generation model may be applied as a low-cost and practical clinical tool that provides powerful auxiliary information for the diagnosis of myocardial blood flow.

## Introduction

Myocardial single-photon emission computed tomography (SPECT) is used worldwide to diagnose myocardial ischemia [1]. Artefacts due to photon absorption significantly affect the specificity of ischemia diagnosis in myocardial SPECT [2]. Therefore, overcoming this well-known artefact is important for improving diagnostic accuracy in myocardial ischemia diagnosis using SPECT. Conventionally, artificial intelligence has been applied in nuclear cardiology to predict obstructive disease on SPECT [3]. Recently, an artificial intelligence-based attenuation correction has been demonstrated [4]. Several attenuation correction approaches were proposed, such as generating attenuation maps (*μ*-maps) from emission images [5, 6] and predicting attenuation-corrected images from non-attenuation-corrected images [7–10]. Both deep-learning models can generate attenuation-corrected SPECT images, eliminating the need for patient radiation exposure by X-ray computed tomography (CT) for attenuation correction. Incidentally, advanced ^13^N ammonia positron emission tomography (PET) has high image quality and precision quantification compared with SPECT. Therefore, PET images were used as references for attenuation-corrected SPECT validation [11]. In other words, PET is currently the best imaging modality with the least artifacts due to photon attenuation in nuclear cardiology. Previously, an image processing approach was proposed for registering SPECT images to PET images based on surface matching [12]. However, this surface-matching approach, which utilized PET images as the gold standard nearly a quarter of a century ago, may not become the current mainstream technique. Focusing on this background of nuclear cardiology with SPECT and PET, we hypothesized that image deep learning using SPECT and PET is potentially useful as a novel approach to correct specific SPECT artefacts. In this study, we proposed the SPECT-to-PET translation model-generated SPECT (SPECT_SPT_) using a deep-learning model of same-patient SPECT and PET datasets. And we mainly assessed the performance of the correction for the attenuation artifacts in the inferior wall. First, we performed voxel-wise analysis of SPECT and SPECT_SPT_ with reference to PET. Second, we analyzed the agreement of SPECT and SPECT_SPT_ representative voxels values in 17 segments according to the American Heart Association (AHA) with reference to PET. Additionally, we compared visual scoring by SPECT and SPECT_SPT_ with PET scores because diagnosis based on visual scoring is still the standard in SPECT, including the summed stress score (SSS), summed rest score (SRS), and summed difference score (SDS) [1, 13].

## Materials and methods

### SPECT and PET datasets

This study was approved by the Institutional Review Board of Tokyo Women’s Medical University (5346) and Kyushu University (2022–127) and was conducted in accordance with the 1964 Declaration of Helsinki and all subsequent revisions. The requirement for written informed consent was waived. SPECT and PET short-axis images of ventricular base-to-apex slices were obtained from 71 patients who underwent rest and stress myocardial ^99m^Tc-methoxyisobutylisonitrile (^99m^Tc-MIBI) SPECT and ^13^N ammonia PET for known or suspected coronary artery disease. ^99m^Tc-MIBI SPECT was performed using a dual-head SPECT/CT gamma-detector scanner (Symbia S; Siemens Healthcare, Erlangen, Germany) equipped with a smart zoom collimator. The acquisition time for each projection was 40 s after stress (approximately 185 MBq, 5 mCi) and 30 s at rest (approximately 555 MBq, 15 mCi). For stress imaging, each patient received an intravenous infusion of adenosine for 6 min (0.12 mg/kg/min), and ^99m^Tc-MIBI was administered 3 min post infusion. Fifteen minutes after the ^99m^Tc-MIBI injection, myocardial perfusion imaging was performed. After a 150-min interval, myocardial perfusion imaging at rest was performed 15 min after the ^99m^Tc-MIBI injection. Thirty projection datasets were obtained in a 128 × 128 matrix over a 180º arc. The reconstructed images were automatically reoriented to the short-axis images. The pixel size of the cine images was 2.4 × 2.4 mm, with a 2.4-mm slice thickness (resolution: 0.4 pixels per mm). ^13^N ammonia PET was performed using a three-dimensional PET system (Biograph mCT; Siemens Healthcare, Erlangen, Germany). Repeatedly upgraded Syngo VA30A_HF07 software was used for dose correction (i.e., the difference in residual ^13^N ammonia activity between resting and stressed images). Sequential CT scans (120 kV, 20 mA, and 3-mm slice collimation) were acquired for attenuation correction. Electrocardiogram-gated image acquisition was performed immediately after the intravenous administration of ^13^N ammonia (approximately 185 MBq, 5 mCi) for 10 min at 16 frames/cardiac cycle using the parallel list mode. After PET myocardial perfusion imaging was performed at rest, the adenosine stress test was performed (0.12 mg/kg/min for 6 min). Three minutes after vasodilator administration, ^13^N ammonia was infused (approximately 555 MBq, 15 mCi), and myocardial perfusion imaging was performed. Images were reconstructed using Fourier re-binning and filtered back-projection with a 12-mm three-dimensional Hann window for the ramp filter. The reconstructed images were automatically reorientated to short-axis images with a 128 × 128 matrix size. The pixel size of the cine images was 1.6 × 1.6 mm, with a 1.6-mm slice thickness (resolution: 0.6 pixels per mm). In other words, the SPECT and PET images were reconstructed into sequential short-axis images with different voxel sizes. Consequently, all patients had a different number of images in the stress and resting states.

### Generating SPECTSPT with deep learning

**Fig. 1:**
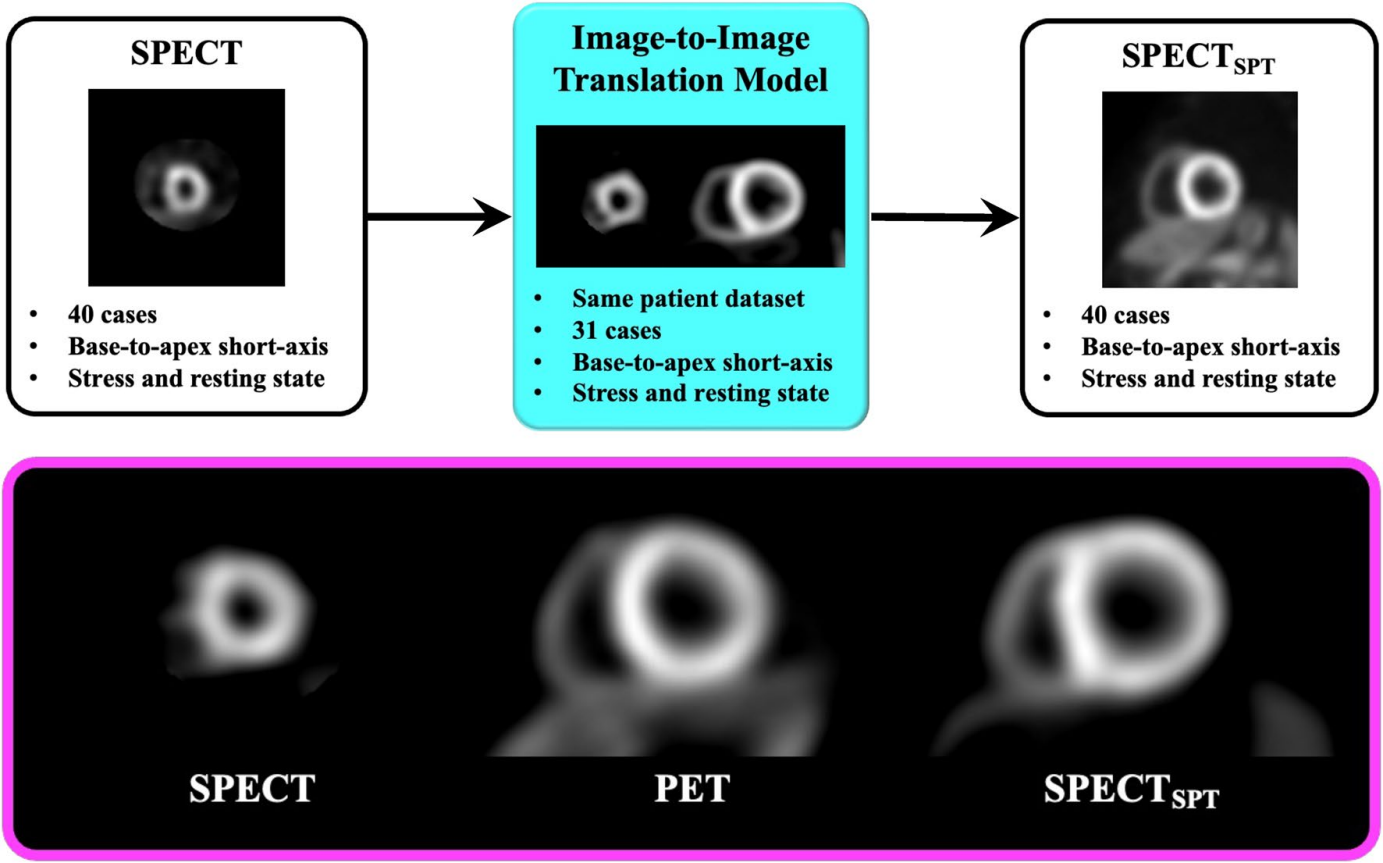
The overall scheme of deep-learning and its representative example. The deep-learning model was trained with 31 SPECT and PET datasets using an image-to-image translation architecture (light blue box). SPECT-to-PET translation model-generated SPECT (SPECT_SPT_) images from nonattenuation-corrected SPECT images (upper panel). The magenta box shows a representative example of the same patient on SPECT (left), PET (middle), and SPECT_SPT_ (right). *SPECT* single-photon emission computed tomography, *PET* positron emission tomography

The training and validation data for generating SPECT_SPT_ were prepared for deep learning in 31 from the 71 cases. Image preprocessing was performed using MATLAB R2020a (version 9.8; MathWorks Inc., Natick, MA, USA) to set patient-to-patient-matched SPECT/PET image datasets. First, SPECT and PET images from the left ventricular base to the apex were selected for all cases. Second, because a different number of base-to-apex images was selected in SPECT and PET owing to the difference in slice thickness, the number of PET slices was aligned with those of SPECT by nearest-neighbor interpolation. Further, the in-plane pixel size of SPECT images was matched to that of PET by image enlargement and cropping. Since the pixel size of PET is 1.6 mm and SPECT is 2.4 mm, SPECT images were magnified 2.4/1.6 times and cropped with a matrix size of 128 × 128, centered at the magnified image. Through this preprocessing, the same number of image datasets from the base to the apex in SPECT and PET were obtained in all cases. The image datasets of 31 cases (stress: 1400 image pairs, rest: 1412 image pairs) were randomly assigned to the training (stress: 979 image pairs, rest: 987 image pairs) and validation (stress: 421 image pairs, rest: 425 image pairs) datasets. Subsequently, the bit depth for all datasets was converted to 8 bits of portable network graphics files. Deep learning was performed using a specialized graphics processing unit (TITAN GeForce1080; Nvidia, Santa Clara, Calif, USA) to create an established image-to-image translation model [14]. The cost function is defined as the summation of two adversarial losses for translating SPECT_SPT_ and SPECT into the other image, along with the cyclic consistency loss for these translations. Consequently, the models for SPT-SPECT generation from SPECT were concreted using 50 epochs with a batch size of 8, a learning rate of 0.002, and a momentum optimizer (Fig. 1).

To test the SPECT_SPT_-generating deep-learning model, a test dataset was prepared using the data of 40 of the 71 cases that were not used for training or validation (stress: 1830 images, rest: 1856 images). For input to the deep-learning model, base-to-apex SPECT images were converted to 8 bits of portable network graphics files with a 256 × 256 matrix size in the same manner as the training and validation datasets. The voxel values of SPECT, PET, and deep learning-derived SPECT_SPT_ images were converted to values of 0‒100, similar to an uptake value, according to the signal value of the 8-bit images (max = 255). Thereafter, the image sizes were downsized to 128 × 128 matrices, the same as the original image (Fig. 1). For voxel-wise analysis of SPECT and SPECT_SPT_ against PET, the normalized root-mean-square-error, peak signal-to-noise ratio, and structural similarity index were quantified.

### Calculation of the voxel values corresponding to the AHA 17-segment model

First, the SPECT, PET, and SPECT_SPT_ sequential short-axis images were longitudinally divided into seven segments. Two segments each were assigned to the basal, middle, and apical regions, and one segment was assigned to the apex. Second, the maximum values of the slice profile were obtained corresponding to the polar coordinates of the 17 AHA segments centered at the apex on the short-axis image. For example, in segment #1 (basal anterior), 60 profiles were set per a short-axis slice. Thus, the number of profiles in segment #1 was 60 multiplied by the number of slices corresponding to the basal region. Finally, the representative voxel values in each segment were calculated as the averaged maximum values of profiles corresponding to each segment.

### Visual evaluation of SPECTSPT with myocardial perfusion scores

Two radiologists and one cardiologist who all had more than 15 years of experience in cardiac nuclear medicine as a routine practice performed consensus scoring of the SPECT, PET, and SPECT_SPT_ myocardial perfusion images of 40 cases. SPECT, PET, and SPECT_SPT_ scans were presented independently in a randomized order, and the evaluators were blinded to all patient information. Sixteen base-to-apex short-axis slices and eight slices in the vertical and horizontal long-axis were displayed as a split image in the stress and resting states (Supplementary Fig. 1). In accordance with the interpretation conditions of the evaluators’ institution, an uptake of 10%‒100% was displayed in RGB-color, no normal database was referred, and zooming and windowing were free. The evaluators determined the stress and resting scores in 17 segments on the SPECT, PET, and SPECT_SPT_ images. Defects were graded according to the regional uptake values as 0 to 4 of 5 scores as normal (75‒100%), mild defect (65‒74%), moderate defect (50‒64%), severe defect (40‒49%), and complete defect (< 40%), respectively. Notably, the uptake value was just the reference value for scoring, and the scores were determined by the evaluators based on their diagnostic experience.

### Statistical analyses

All statistical analyses were performed using MATLAB with the Statistics and Machine Learning Toolbox R2020a (version 9.8; MathWorks Inc., Natick, MA, USA). Differences were considered statistically significant at a *P*-value < 0.05. Normally distributed data are presented as the mean ± standard deviation and non-normally distributed data are presented as the median and interquartile range (IQR, 25th–75th percentile). Based on the data distributions provided by the Shapiro–Wilk test [15], patient characteristics and ^13^N ammonia PET measurements between the training and validation groups and test groups were compared using the unpaired *t*-test or Mann–Whitney U test. The normalized root-mean-square-error, peak signal-to-noise ratio, and structural similarity index against PET were compared between SPECT and SPECT_SPT_ by the paired *t*-test or Wilcoxon matched-pairs signed rank test. The representative voxel values of SPECT and SPECT_SPT_ in the 17 AHA segments against those of PET were compared using the paired *t*-test or Wilcoxon matched-pairs signed rank test. Further, the errors of representative 17-segment voxel values of SPECT and SPECT_SPT_ against PET were also compared using the paired *t*-test or Wilcoxon matched-pairs signed rank test. The stress, resting, and difference scores of 40 test cases of SPECT and SPECT_SPT_ were compared to those of PET by the paired *t*-test or Wilcoxon matched-pairs signed rank test in each of the 17 AHA segments. The diagnostic ability of the presence of a PET defect area in the segments of the right coronary artery (RCA), left anterior descending artery (LAD), and left circumflex artery (LCX) using SPECT scores of SSS, SRS, and SDS in each segment were analyzed by receiver operating characteristic (ROC) analysis. The presence of a PET defect was defined as one or more SSS, SRS, or SDS in each coronary segment. Additionally, the diagnostic ability of the presence of a PET defect area using both SPECT and SPECT_SPT_ scores was also analyzed by ROC analysis.

## Results

Sixty-four of the 71 cases underwent SPECT examination followed by PET examination. Twenty-six patients underwent SPECT and PET follow-up scans within 4 months because they were clinically stable. In 14 patients, a PET scan was performed at the latest 1 month after SPECT due to difficulty in distinguishing between ischemia and attenuation artifacts by SPECT. A total of 40 patients [14 and 26] were assigned to be test cases for deep learning. The remaining 31 patients were assigned as training cases who were scanned SPECT and PET within 3 years; however, we carefully confirmed that there were no clinical changes between the imaging sessions. The absolute duration between SPECT and PET examinations in training cases was 415 (331–599) days and in test cases was 46 ± 31 days. Table 1 compares the characteristics between the training (*n* = 31) and test (*n* = 40) groups. Although the percentage of cases with obesity was slightly higher in the training group than in the test group, no significant differences were found in most patient background characteristics, cardiovascular risk factors, and clinical history information between the training and test groups. The stressed end-diastole ventricular volume index and ejection fraction in the stress and resting states in the test group were lower than those in the training group, however, there were no differences between the two groups in terms of the percentage of the ventricular dilation compared with the previous normal value [16].

**Table 1:** Patient characteristics and N-13 ammonia PET measurements

	Training (*n* = 31)	Test (*n* = 40)	*P* value
Baseline characteristics			
Male, *n* (%)	22 (71)	32 (80)	0.38
Age (years)	69 ± 9	65 ± 11	0.14
Height (cm)	163 ± 9	164 ± 7	0.51
Weight (kg)	65 ± 13	64 ± 11	0.60
BMI (kg/m^2^)	24.6 ± 4.1	23.7 ± 3.3	0.29
Obesity (BMI > 25)	15 (48%)	10 (25%)	0.04
Cardiovascular risk factor			
Anemia (Male: Hb < 13, Female: Hb < 11)	8 (26%)	10 (25%)	0.94
Hypertension	20 (65%)	30 (75%)	0.34
Dyslipidemia	17 (55%)	26 (65%)	0.39
Diabetes mellitus	11 (35%)	22 (55%)	0.11
Smoking	10 (32%)	15 (41%)	0.65
Clinical history			
RCA stenosis	5 (16%)	13 (33%)	0.12
LAD stenosis	8 (26%)	12 (30%)	0.70
LCX stenosis	7 (23%)	9 (23%)	> 0.99
Previous PCI	8 (26%)	15 (38%)	0.30
Previous CABG	3 (10%)	3 (8%)	0.75
Ammonia PET measurements			
Stressed EDVi (mL/m^2^)	58 (53–71)	69 ± 17	0.29
Ventricular dilation (EDVi > 70)	10 (32%)	21 (53%)	0.76
Resting EDVi (mL/m^2^)	49 (44–64)	59 ± 17	0.12
Ventricular dilation (EDVi > 70)	6 (19%)	9 (23%)	0.07
Stressed ESVi (mL/m^2^)	18 (13–31)	28 ± 15	0.03
Ventricular dilation (ESVi > 25)	9 (29%)	22 (55%)	0.08
Resting ESVi (mL/m^2^)	13 (9–24)	24 ± 14	0.06
Ventricular dilation (ESVi > 25)	7 (23%)	16 (40%)	0.12
Stressed EF (%)	68 ± 11	61 ± 13	0.02
Resting EF (%)	74 (63–80)	64 ± 13	0.04
Stressed MBF (mL/g/min)	2.0 ± 0.5	1.8 ± 0.4	0.06
Resting MBF (mL/g/min)	0.9 (0.8–1.2)	1.0 ± 0.2	0.80
Global MFR	2.2 ± 0.5	2.0 ± 0.6	0.12

Normal distributed data are presented as the mean ± standard deviation. Non-normal distributed data are presented as the median and interquartile range (IQR, 25th–75th percentile) with an underline

*EDVi* end-diastolic volume index, *ESVi* end-systole volume index, *EF* ejection fraction, *MBF* myocardial blood flow, *MFR* myocardial flow reserve

**Fig. 2:**
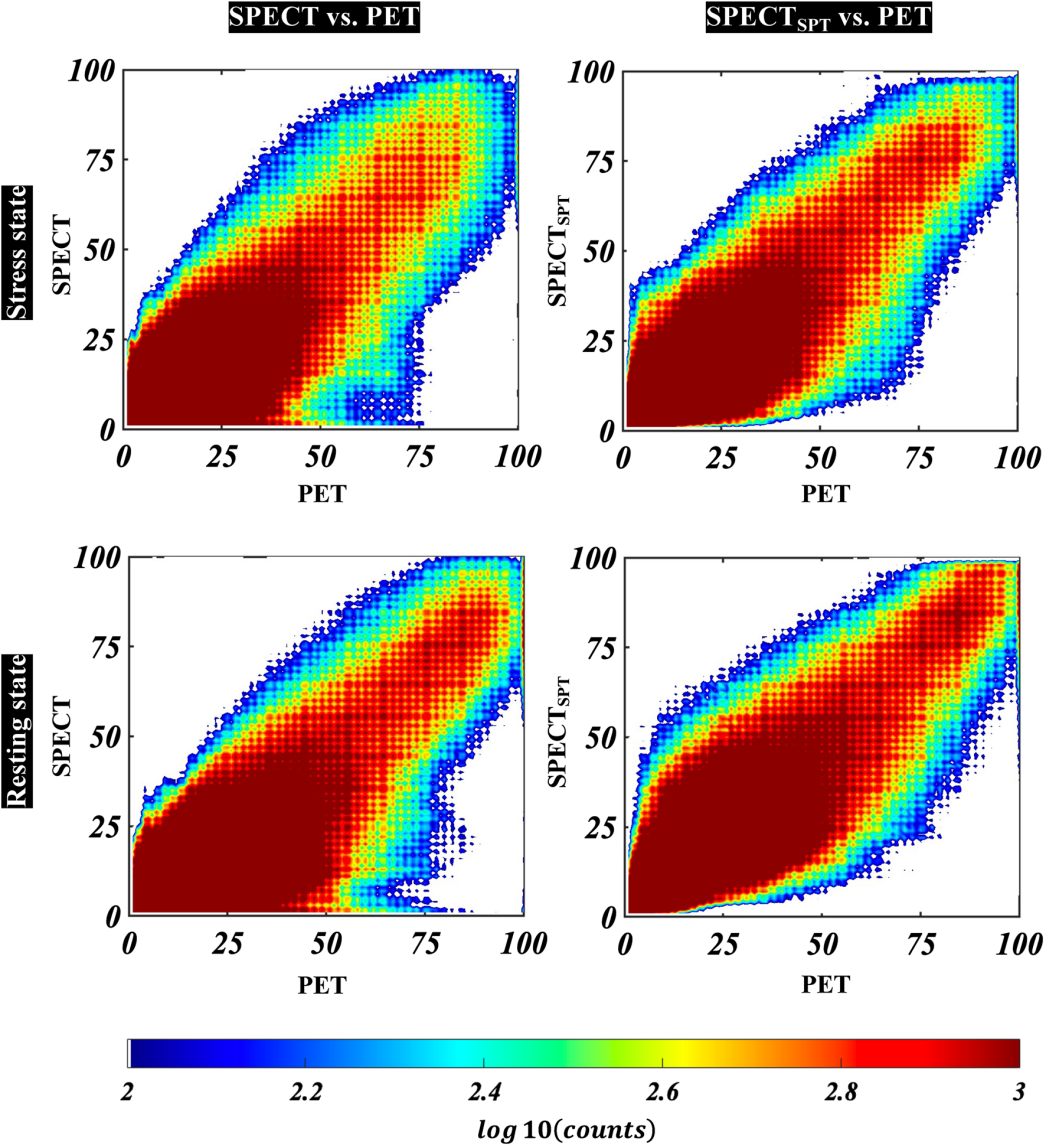
Joint histograms that represent the voxel values of SPECT and SPECT_SPT_ corresponding to PET. Left row plots indicate PET voxel values (*x*) and SPECT voxel values (*y*) at stress (upper) and resting states (lower). Right row plots indicate PET voxel values (*x*) and SPECT_SPT_ voxel values (*y*) at stress (upper) and resting (lower) states. Counts of voxel were log10-scaled to color visualization in joint histograms. SPECT_SPT_, SPECT-to-PET translation model-generated SPECT; SPECT, single-photon emission computed tomography; PET, positron emission tomography

For voxel-wise analysis, the normalized root mean square errors of SPECT and SPECT_SPT_ with respect to PET were 0.032 ± 0.005 and 0.030 ± 0.006 in the stress state (*P* = 0.005) and 0.027 ± 0.005 and 0.035 ± 0.006 (*P* < 0.0001) in the resting state, respectively. The respective peak signal-to-noise ratios were 17.1 ± 1.5 and 18.6 ± 1.5 (*P* < 0.0001) and 16.4 ± 1.6 and 18.1 ± 1.4 (*P* < 0.01) and the respective structural similarity indices were 0.613 ± 0.039 and 0.719 ± 0.044 (*P* < 0.0001) and 0.621 ± 0.039 and 0.723 ± 0.042 (*P* < 0.0001), respectively. Figure 2 shows joint histograms that represent the voxel values of SPECT or SPECT_SPT_ corresponding to PET in the testing group with a scatter plot and count of plots. The under-voxel-values of less than 25 of SPECT (but 50 and 75 of PET) were improved in SPECT_SPT_ in both stress and resting states.

**Fig. 3:**
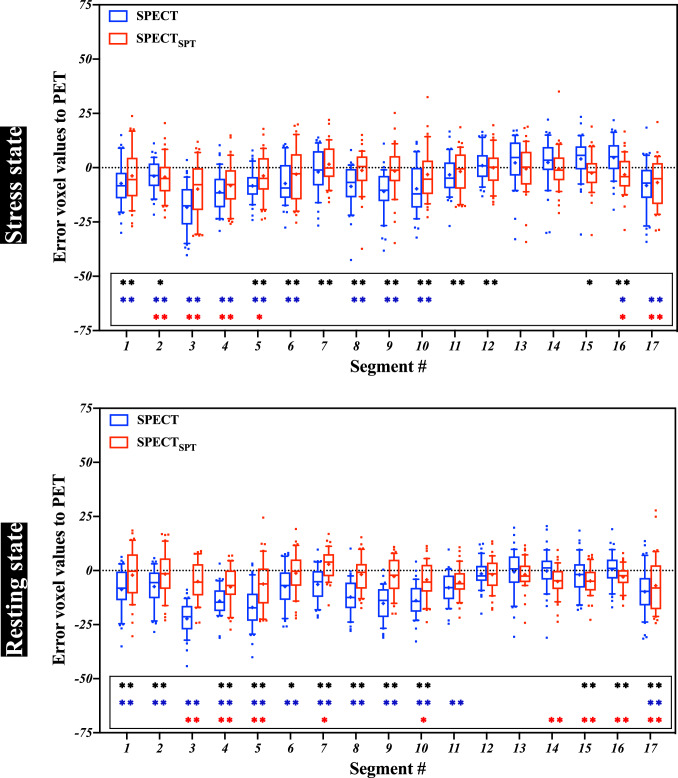
AHA 17-segment-wise box-and-whisker plots of the error voxel values with respect to PET in 40 test patients. Upper and lower plots are indicated at stress and resting states, respectively. The blue boxes represent the differences between the voxel value of SPECT and PET and the red boxes represent those of SPECT_SPT_. The lines at the ends of the boxes indicate median values. The symbol “ + ” indicates the mean value. The blue and red asterisks indicate significant differences in voxel values from SPECT and SPECT_SPT_ against PET. The black asterisk indicates a significant difference in error between SPECT and SPECT_SPT_ against PET. AHA, American Heart Association; SPECT_SPT_, SPECT-to-PET translation model-generated SPECT; *SPECT* single-photon emission computed tomography, *PET* positron emission tomography; **, *P* < 0.01; *, *P* < 0.05

For AHA 17-segment-wise analysis, box-and-whisker plots of the errors of representative voxel values against PET in 40 test patients are shown in Fig. 3. In the stress state, we observed significant errors in SPECT voxel values at basal anterior regions (segments #1, #6), and at mid inferoseptal regions (segments #8, #9, and #10) but not in SPECT_SPT_. In the resting state, we observed significant errors in SPECT voxel values at basal anterior regions (segments #1, #2, and #6), and at mid-inferior regions (segments #8, #9, and #11). but not in SPECT_SPT_. Further, at inferior regions in the base and middle of the ventricle (segments #4, #5, and #10), both SPECT and SPECT_SPT_ had significant errors with respect to PET; however, SPECT_SPT_ errors were smaller than SPECT. Errors were observed in SPECT_SPT_ voxel values in segments at apical regions (#14, #15, and #16) in the resting state but not in SPECT_SPT_.

**Fig. 4:**
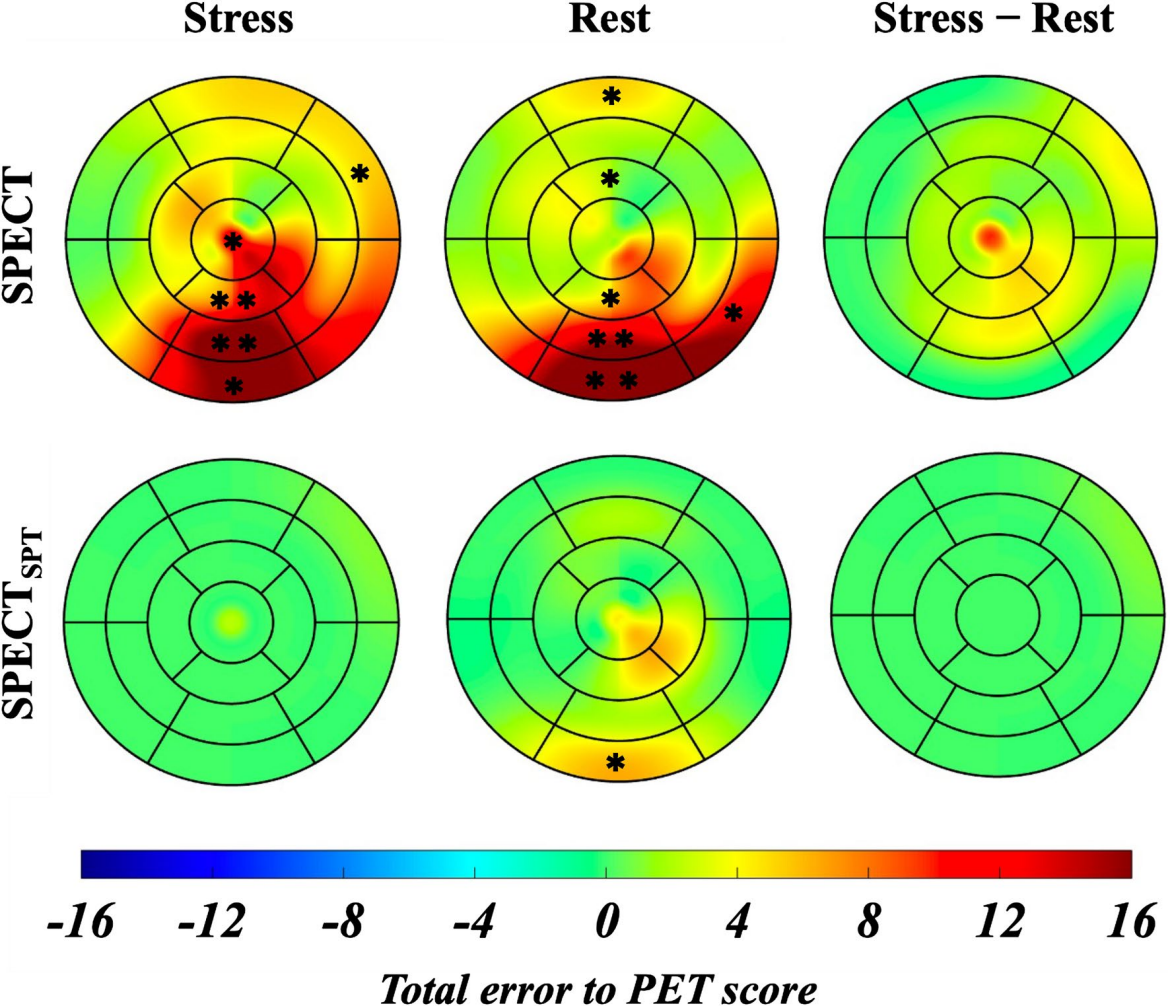
Colored polar map of the total error of 40 test cases of SPECT and SPECTSPT myocardial perfusion scores against PET in 17 segments. Blue, green, and red indicate the underestimated, equivalent, and overestimated mean error in the myocardial perfusion scores compared with those of PET, respectively. The asterisks indicate that the SPECT and SPECT_SPT_ scores are significantly different from those of PET. SPECT_SPT_, SPECT-to-PET translation model-generated SPECT; *SPECT* single-photon emission computed tomography, *PET* positron emission tomography; **, *P* < 0.01; *, *P* < 0.05

**Fig. 5:**
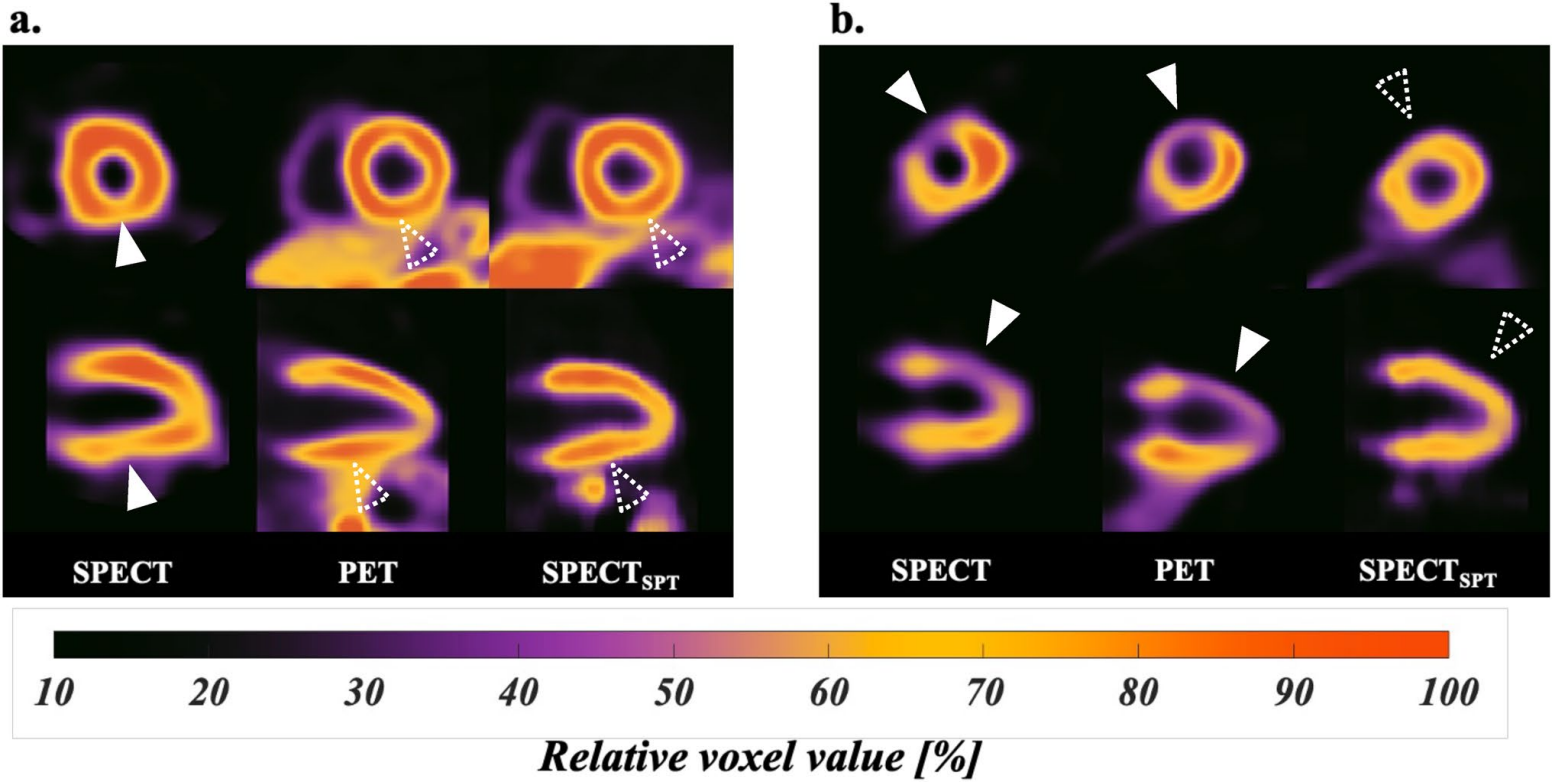
The same patient cases of SPECT, PET, and attenuation-corrected SPECT_SPT_. **a** Short-axis and vertical long-axis colored relative uptake images of a 54-year-old man with ventricular dilation (EDVi = 122 mL/m^2^ and ESVi = 74 mL/m^2^) but without ischemia are shown. White arrowheads indicate slightly reduced activity in the mid-inferior region on SPECT, but no reduced activity areas were found using PET and SPECT_SPT_ (white dashed line arrowheads). **b** Short-axis and vertical long-axis colored relative uptake images of a 50-year-old man with ischemia are shown. Significant middle to apical defect areas were observed on SPECT and PET, but the defect areas disappeared on SPECT_SPT_. EDVi, end-diastole volume index; ESVi, end-systole volume index; SPECT_SPT_, SPECT-to-PET translation model-generated SPECT; SPECT, single-photon emission computed tomography; PET, positron emission tomography

Figure 4 shows a colored polar map of the total error of 40 test cases of myocardial perfusion scores of SPECT and SPECT_SPT_ against those of PET in 17 segments. Compared with the PET stress and resting scores, significantly overscoring of the SPECT stress scores at segments at inferior regions from base-to-apex (#4, #10, and #15) was observed. In contrast, no significant overscores were observed in SPECT_SPT_ during stress, and only a moderate overscore and moderate underscore at the basal inferior region of segment #4 in the resting and difference states were observed, respectively. The presence of PET defect areas in the RCA, LAD, and LCX segments were diagnosed by SPECT summed scores with area under the curves of ROC (78%, 68%, and 78%), sensitivities (53%, 33%, and 33%), and specificities (96%, 100%, and 100%), respectively. Similarly, the diagnostic abilities for a PET defect area by SPECT and SPECT_SPT_ summed scores were area under the curves of ROC (85%, 73%, and 89%), sensitivities (54%, 33%, and 56%), and specificities (100%, 100%, and 100%), respectively. Figure 5a (left) shows representative images of SPECT, PET, and attenuation-corrected SPECT_SPT_ of the same patient. The low accumulation in the middle inferolateral region on SPECT was corrected using SPECT_SPT_. Figure 5b (right) shows bad correction in SPECT_SPT_ as the disappeared apical anterior defect area, whereas the defect areas were observed by SPECT and PET.

## Discussion

According to the voxel-wise analyses, significant differences between the normalized root-mean-square-error of SPECT and SPECT_SPT_ with respect to PET were observed at the stress and resting states. However, the value of normalized root-mean-square-errors from both SPECT and SPECT_SPT_ are small compared to previous reports [8]. Consequently, the SPECT, PET, and SPECT_SPT_ images can be considered globally consistent. In the image similarity analysis by peak signal-to-noise ratio and the structural similarity index, slight but statistically significant increases in similarity indices in SPECT_SPT_ rather than SPECT were observed at the stress and resting states. The similarity indices were lower than those observed in recent research on attenuation-corrected SPECT generation from SPECT with deep learning [9]. However, this finding is reasonable because in this study only SPECT and PET images were compared. Therefore, the SPECT and SPECT_SPT_ images can be considered strictly different with reference to PET images. Comparison of the joint histograms of both stress and resting states revealed that under-voxel-values in SPECT are improved in SPECT_SPT_. According to the AHA segment-wise analysis, the representative voxel values of SPECT at mid-inferoseptal regions (segments #8, #9, and #10) were significantly smaller than PET, but there was no difference between PET and SPECT_SPT_. Further, a comparison of resting voxel values at basal inferior regions (segments #4 and #5) revealed that the representative voxel values of SPECT and SPECT_SPT_ against PET were both significantly smaller than PET, but the errors of SPECT_SPT_ were significantly smaller than those of SPECT. In the comparison of the mean error to PET visual scores in SPECT and SPECT_SPT_, we observed significant overscoring at the inferior wall at base-to-apex wall in SPECT were mostly corrected in SPECT_SPT_ as equal as PET. The image artifact in SPECT at the inferior wall is a well-known result of photon attenuation. It is also well-known that prone images improve attenuation correction in the anterior, anteroseptal, lateral, and inferior areas [17]. Although prone imaging is a simple and useful clinical technique that does not require complex image reconstruction algorithms or software, it increases examination time due to the additional scan. Since prone position SPECT and PET data could not be used in this study, our inability to directly compare them is considered a study limitation. However, we believe that our PET-supervised deep learning model is a new approach to correct well-known attenuation in SPECT myocardial perfusion imaging without the need for an additional scan. While SPECT_SPT_ was effective in correcting attenuation in the inferior region, the disappearance of true defects in the anterior region due to overcorrection was also observed. We acknowledge that the disappearance of defects is a weakness of the estimation using deep learning. In this study, we did not label the training images with “true defect” or “false defect due to the attenuation”. The error of activity disappearance may be improved by adding meta information to the training images. However, the area under the curves for detecting the presence of PET defect areas in the RCA, LAD, and LCX segments tended to improve using summed scores with both SPECT and SPECT_SPT_ than with only SPECT. Consequently, SPECT_SPT_ is expected to be applied in clinical situations to provide additional diagnostic support information in myocardial perfusion imaging but it is not an alternative imaging modality to SPECT.

In recent years, deep learning has been reported to generate SPECT images with CT attenuation correction from SPECT without attenuation correction [8, 18]. Conversely, dedicated cardiac SPECT scanners with cadmium-zinc-telluride (CZT) detectors are clinically available [19]. Since dedicated cardiac SPECT scanners are designed compactly without CT, attenuation correction with deep learning based on attenuation-corrected SPECT as a training dataset is challenging [20]. Myocardial PET has become widely available for the quantitative assessment of myocardial perfusion [21–24]. This proposed method may be promising to apply to CT-less SPECT imaging by preparing patient-to-patient datasets of PET and SPECT images. Furthermore, similar to the CT-less deep-learning approaches, our proposed CT-less method reduces radiation exposure. Our SPECT_SPT_ generation method is a novel approach to strengthen the clinical usefulness of SPECT in nuclear cardiology.

We acknowledge some limitations of this study. First, this study had a small sample size from a single center. This limitation might be due to only a few institutions in our country, which have the required ^13^N ammonia production. Since the number of images in the training set used for image generation was over 1000, it was considered sufficient for building an image-to-image translation model. However, model rebuilding and validation in larger and more diverse cohorts is essential. Second, only a few patients (*n* = 14) underwent a PET scan within 1 month due to the difficulty of distinguishing between ischemia and attenuation artifacts by SPECT. It is necessary to obtain a large number of cases who underwent PET within one month due to an uncertain SPECT diagnosis and use them for both deep learning training and testing. Therefore, we plan to conduct a multicenter study and undertake further model refinement and validation. Third, both the training and testing groups had an insufficient and biased number of cases of obesity, RCA stenosis, and ventricular dilation, which are likely to produce well-known SPECT artifacts. Although, in this study cohort, there was no difference in most clinical characteristics between the training and test groups, the cohort were considered that attenuation artifacts are patient-specific and affected by characteristics such as sex, obesity, and ventricular size. We used an image-to-image translation network developed in a previous study [14]. U-Net network was used as the generator for this network [25]. The performance of the proposed model may be improved in the future by incorporating metadata such as patient height, weight, and the presence or absence of coronary stenosis into the down-sampling layer of U-Net as a numerical matrix [26]. Since this was the first attempt to translate SPECT images with PET as the supervisor, we needed to determine whether this deep learning approach worked well for attenuation correction using the simplest examinations possible. Further, accurate metadata were not always available in retrospective studies. We aim to improve our proposed SPECT-to-PET translation model in the near future.

In conclusion, this study is the first to generate PET-like images from SPECT images based on deep learning. We believe that our PET-supervised deep learning model is a new approach to correct well-known inferior wall attenuation in SPECT myocardial perfusion imaging. Considering PET as the reference, our proposed SPECT_SPT_ is consistent with SPECT, but strictly different in terms of the AHA 17-segment model. This proposed method is a post-processing deep-learning model that provides PET-like image information from CT-less SPECT images. Since standalone SPECT systems are used worldwide, the SPECT_SPT_ generation model may be applied as a low-cost and practical clinical tool that provides powerful auxiliary information for myocardial blood flow diagnosis.

## Data Availability

Data from this research cannot be used for secondary purposes.

## Abbreviations

SPECT: Single-photon emission computed tomography
CT: Computed tomography
PET: Positron emission tomography
SPECT_SPT_: SPECT-to-PET translation model-generated SPECT
AHA: American Heart Association
SSS: Summed stress score
SRS: Summed rest score
SDS: Summed difference score
^99m^Tc-MIBI: ^99M^Tc-methoxyisobutylisonitrile
RCA: Right coronary artery
LAD: Left anterior descending artery
LCX: Left circumflex artery
ROC: Receiver operating characteristic
CZT: Cadmium-zinc-telluride
